# Supplementary material for: Clinical relevance of molecular characteristics in Burkitt lymphoma differs according to age
Source: Nat Commun. 2022 Jul 6;13:3881. doi: 10.1038/s41467-022-31355-8 (PMC9259584; doi:10.1038/s41467-022-31355-8)
Supplement: Supplementary file 1 — Supplementary Information [file 41467_2022_31355_MOESM1_ESM.pdf]

Supplementary Information for

Clinical relevance of molecular characteristics in Burkitt lymphoma over age groups

Birgit Burkhardt*ƚ[1], Ulf Michgehl*[1], Jonas Rohde*[1], Tabea Erdmann[2], Philipp Berning[2], Katrin Reutter[1], Marius Rohde[3], Arndt Borkhardt[4], Thomas Burmeister[5], Sandeep Dave[6], Alexandar Tzankov[7], Martin Dugas[8], Sarah Sandmann[9], Falko Fend[10], Jasmin Finger[1], Stephanie Mueller[1], Nicola Gökbuget[11], Torsten Haferlach[12], Wolfgang Kern[12], Wolfgang Hartmann[13], Wolfram Klapper[14], Ilske Oschlies[14], Julia Richter[14], Udo Kontny[15], Mathias Lutz[16], Britta Maecker-Kolhoff[17], German Ott[18], Andreas Rosenwald[19], Reiner Siebert[20], Arend von Stackelberg[21], Brigitte Strahm[22], Wilhelm Woessmann[23], Martin Zimmermann[17], Myroslav Zapukhlyak[2], Michael Grau[+2], Georg Lenz[+2]

[1] Pediatric Hematology, Oncology and BMT, University Hospital Münster, Münster, Germany
[2] Department of Medicine A, Hematology, Oncology, and Pneumology, University Hospital Münster, Münster, Germany
[3] Pediatric Hematology and Oncology, University Hospital Giessen, Giessen, Germany
[4] Department of Pediatric Oncology, Hematology and Clinical Immunology, University Children's Hospital Medical Faculty, Heinrich-Heine-University, Düsseldorf, Germany
[5] Department for Hematology, Oncology and Tumor Immunology, Corporate Member of Freie Universität Berlin and Humboldt-Universität zu Berlin, Charité - Universitätsmedizin Berlin, Berlin, Germany
[6] Center for Genomic and Computational Biology and Department of Medicine, Duke University, Durham, NC
[7] Institute of Medical Genetics and Pathology, University Hospital Basel, University of Basel, Basel, Switzerland
[8] Institute of Medical Informatics, Heidelberg University Hospital, Heidelberg, Germany
[9] Institute of Medical Informatics, University of Münster, Münster, Germany
[10] Institute of Pathology and Neuropathology and Comprehensive Cancer Centre Tübingen, University Hospital Tübingen, Eberhard-Karls-University, Tübingen, Germany
[11] Department of Medicine II, Goethe University, Frankfurt, Germany
[12] MLL Munich Leukemia Laboratory, Munich, Germany
[13] Gerhard-Domagk-Institute for Pathology, University Hospital of Muenster, Muenster, Germany
[14] Department of Pathology, Hematopathology Section, University Hospital Schleswig-Holstein, Kiel, Germany
[15] Section of Pediatric Hematology, Oncology, and Stem Cell Transplantation, Department of Pediatric and Adolescent Medicine, RWTH Aachen University Hospital, Aachen, Germany
[16] Department of Hematology and Oncology, University Hospital Augsburg, Augsburg, Germany
[17] Hannover Medical School, Department of Pediatric Hematology and Oncology, Hannover, Germany
[18] Department of Clinical Pathology, Robert-Bosch-Krankenhaus, and Dr. Margarete Fischer-Bosch Institute of Clinical Pharmacology, Stuttgart, Germany
[19] Institute of Pathology, Universität Würzburg and Comprehensive Cancer Centre Mainfranken (CCCMF), Würzburg, Germany
[20] Institute of Human Genetics, Ulm University and Ulm University Medical Center, Ulm, Germany
[21] Department of Pediatric Oncology Hematology, Charité - Universitätsmedizin Berlin, Berlin, Germany
[22] Department of Pediatrics and Adolescent Medicine Division of Pediatric Hematology and Oncology, Medical Center Faculty of Medicine, University of Freiburg, Freiburg im Breisgau, Germany
[23] Pediatric Hematology and Oncology, University Medical Centre Hamburg-Eppendorf (UKE), Hamburg, Germany

[*] Co-first author (contributed equally)
[ƚ] Corresponding author
[+] Co-last author (contributed equally)

Table of Contents

Supplementary Figures **2**
Supplementary Fig. 1: Schematic overview of the analysis pipeline 2
Supplementary Fig. 2: Mutational profile of mature B-cell lines 3
Supplementary Fig. 3: Mutational spectrum of MYC 4
Supplementary Fig. 4a,b: Overlap of recurrent mutations in ID3 (C), CCND3 (D) and TCF3 (E) 5
Supplementary Fig. 4c: Mutational spectrum of ID3 6
Supplementary Fig. 4d: Mutational spectrum of CCND3 7
Supplementary Fig. 4e: Mutational spectrum of TCF3 8
Supplementary Fig. 5a,b: Overlap of recurrent mutations of the SWI/SNF complex members ARID1A and SMARCA4 9
Supplementary Fig. 5c: Mutational spectrum of ARID1A 10
Supplementary Fig. 5d: Mutational spectrum of SMARCA4 11
Supplementary Fig. 6a: Mutational spectrum of DDX3X 12
Supplementary Fig. 6b: Mutational spectrum of GNA13 13
Supplementary Fig. 6c,d: Overlap of recurrent mutations of GNA13 and P2RY8 14
Supplementary Fig. 6e: Mutational spectrum of P2RY8 15
Supplementary Fig. 7: Additional associations with outcome in pediatric BL 16
Supplementary Fig. 8: Overview of available primary BL samples and measurements 17
Supplementary Tables **18**
Supplementary Table 1: Overview and outline of clinical characteristics of the pediatric cohort. 18
Supplementary Table 2: Source centers of analyzed samples. 19
Supplementary Table 3: Overview of regulated processes and molecular functions from gene ontology 21
Supplementary Table 4: Selected 134 genes for targeted sequencing 22
Supplementary Table 5: Methods, tools, resources and software used in this study 23
Supplementary Table 6: Selected primers used for Sanger sequencing 25
List of Supplementary Data **26**
Supplementary Data 1: Cohort overview 26
Supplementary Data 2: Called somatic mutations (summarized by Fig.1). 26
Supplementary Data 3: Variant filtering overview and statistics of the multi-stage filter hierarchy 26
Supplementary Data 4: Validation of discovered somatic mutations by Sanger sequencing 26
Supplementary Data 5: Identification of cancer genes by mutation abundance using dN/dS 26
Supplementary Data 6: Discovered recurrent somatic copy number aberrations (GISTIC analysis) 26
Supplementary Data 7: Discovered copy number aberrations on gene level (ASCAT analysis) 26
Supplementary Data 8: Subcohort comparisons (summarized by Figs. 3 and 4). 26
Supplementary Data 9: Hotspot analysis summary for pediatric and adult samples 26

Supplementary Figures

Supplementary Fig. 1: Schematic overview of the analysis pipeline

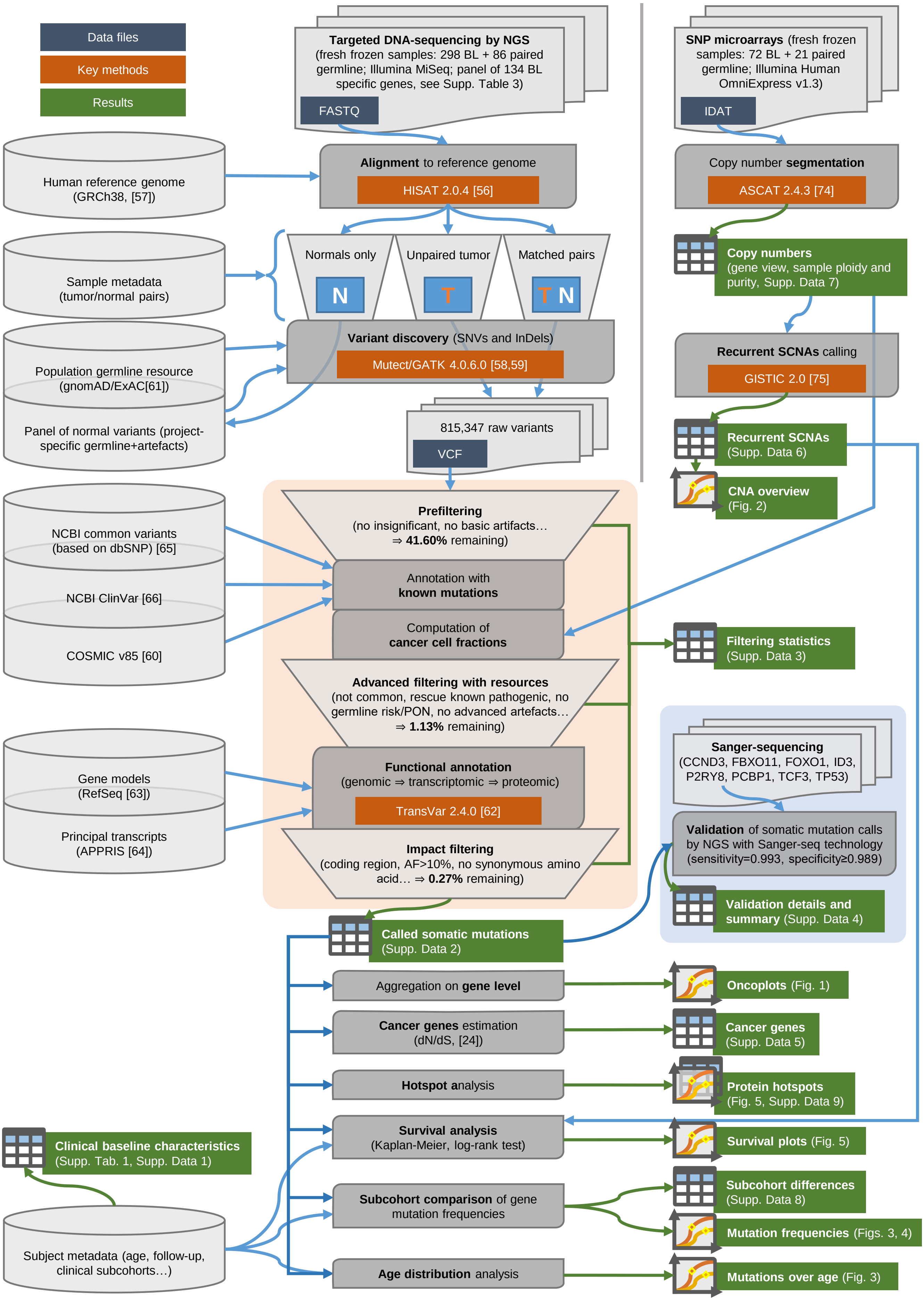

Supplementary Fig. 2: Mutational profile of mature B-cell lines

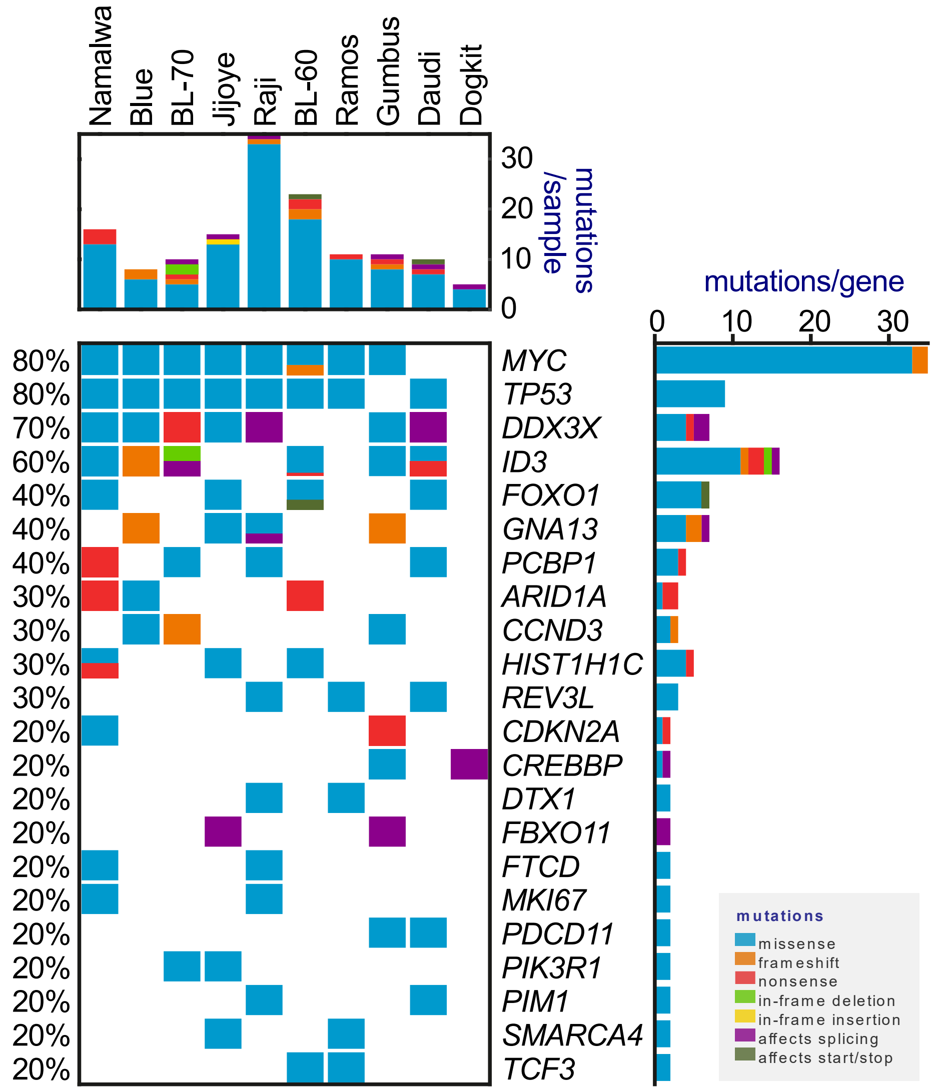

These cell lines served as positive controls of the analysis pipeline and might help select appropriate models for future experiments.

Supplementary Fig. 3: Mutational spectrum of MYC

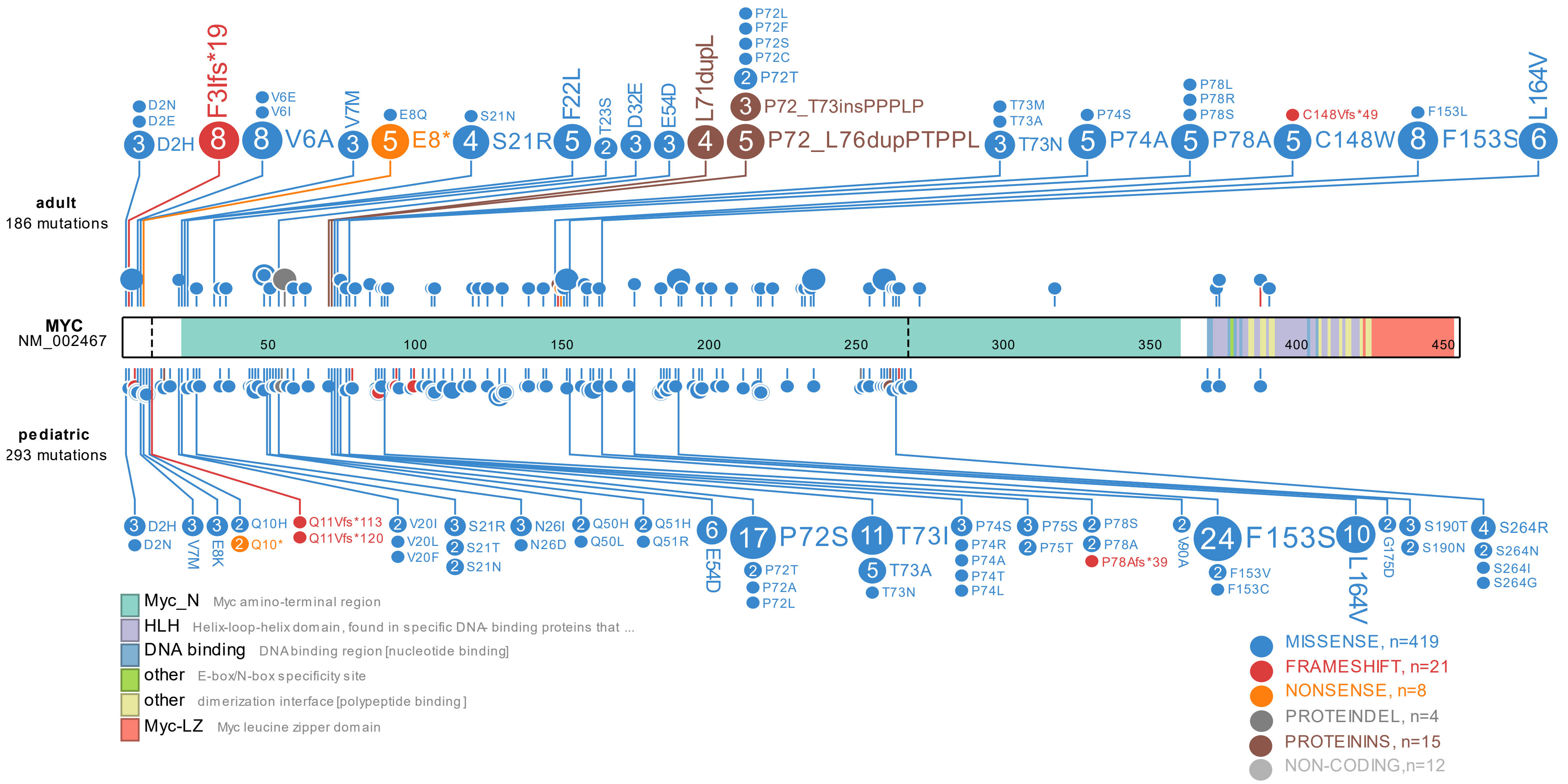

Mutations of MYC in adult patients are shown above the protein axis, pediatric cases below. Differences between age groups include F153S (pediatric: 13% vs adult: 8%), P72S (9%/1%), F3Ifs*19 (0%/8%), V6A (0%/8%) and similarities: E54D (3%/3%), L164V (5%/6%).

Supplementary Fig. 4a,b: Overlap of recurrent mutations in ID3 (C), CCND3 (D) and TCF3 (E)

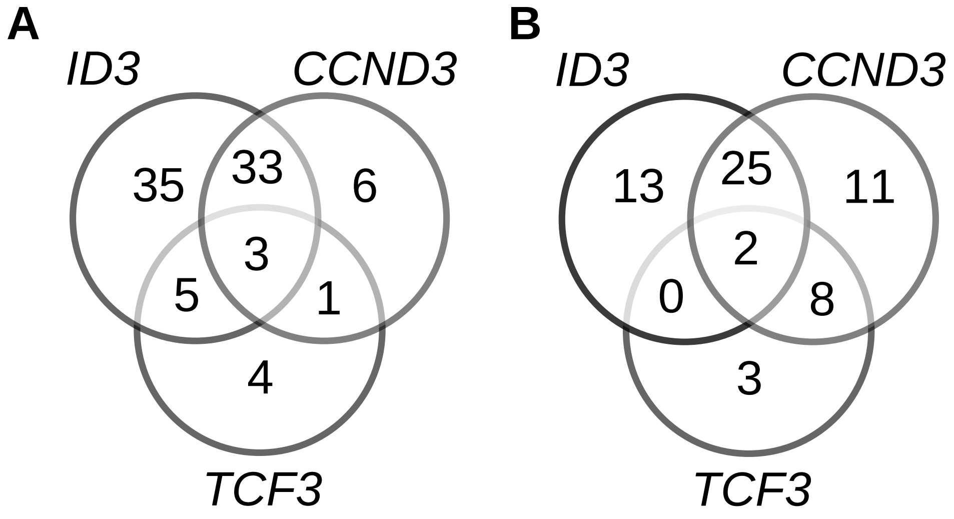

The overlap of recurrent mutations in *ID3*, *CCND3* and *TCF3* is shown for the pediatric (A) and adult subcohort (B) in percent. Taken together, 87% of pediatric cases versus only 63% of adult cases are mutated in any of these genes (p=2e-6, one-tailed Fisher exact test).

Supplementary Fig. 4c: Mutational spectrum of ID3

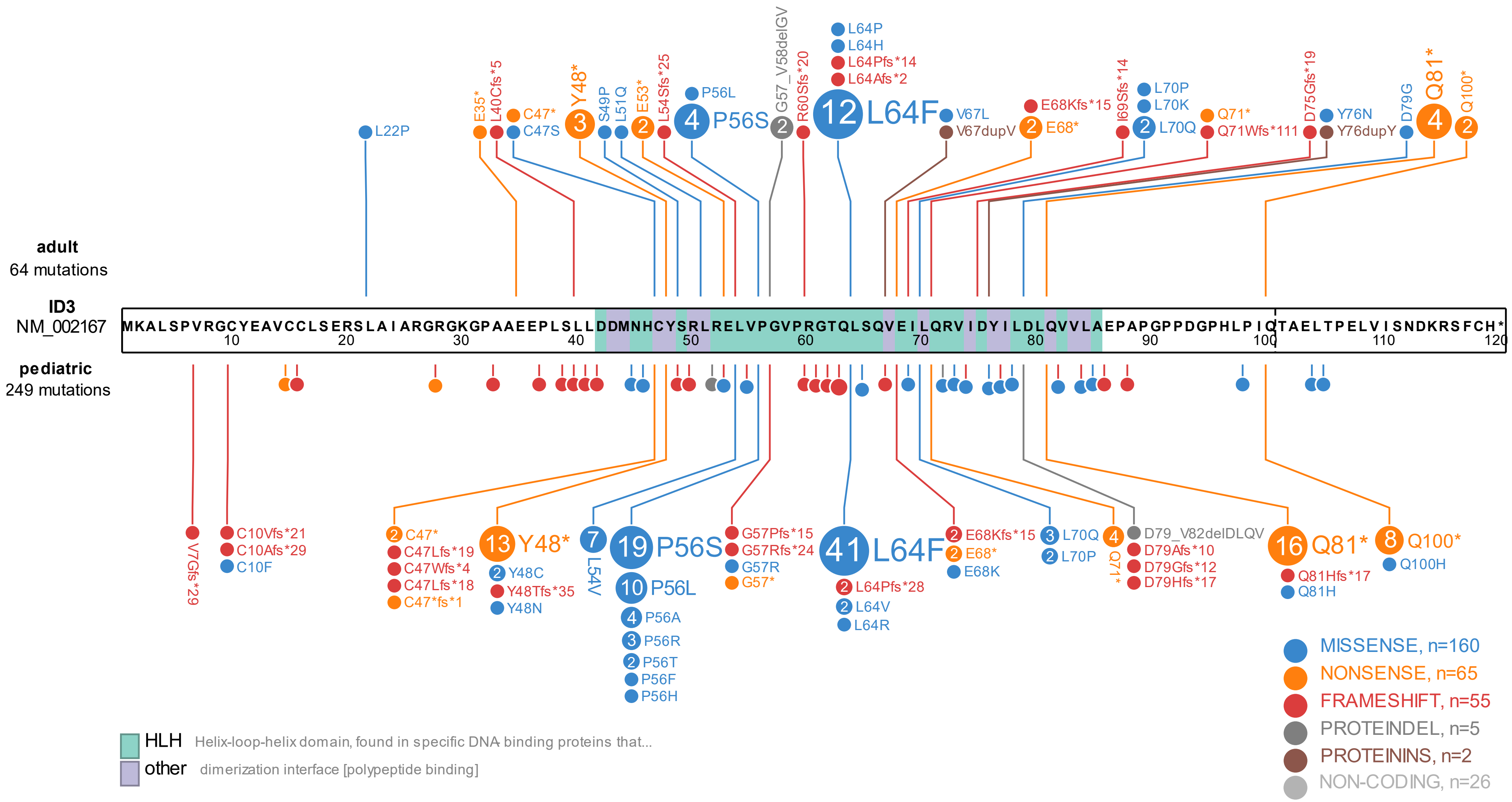

Mutations of ID3 in adult patients are shown above the protein axis, pediatric cases below.

Supplementary Fig. 4d: Mutational spectrum of CCND3

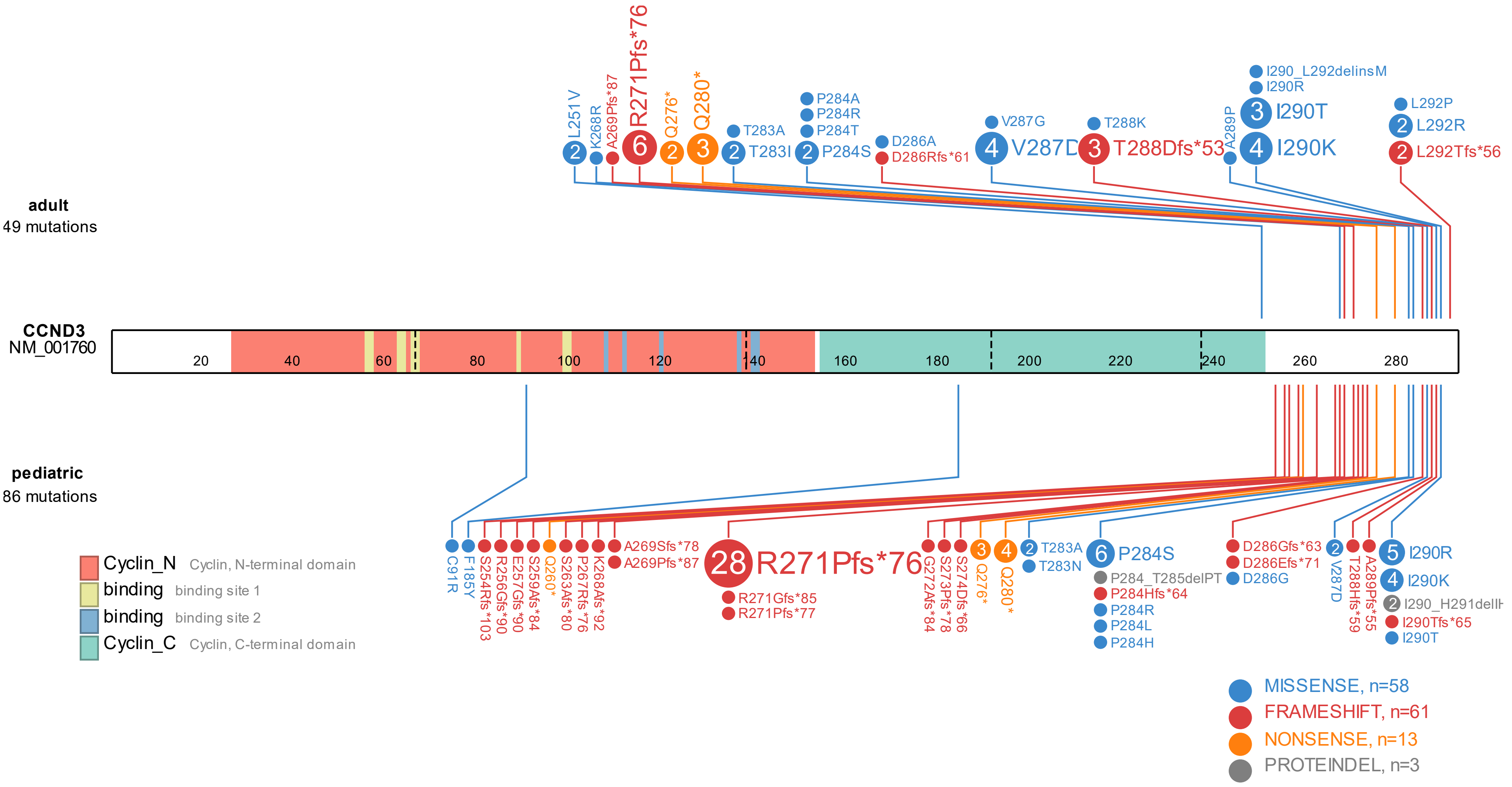

Mutations of CCND3 in adult patients are shown above the protein axis, pediatric cases below.

Supplementary Fig. 4e: Mutational spectrum of TCF3

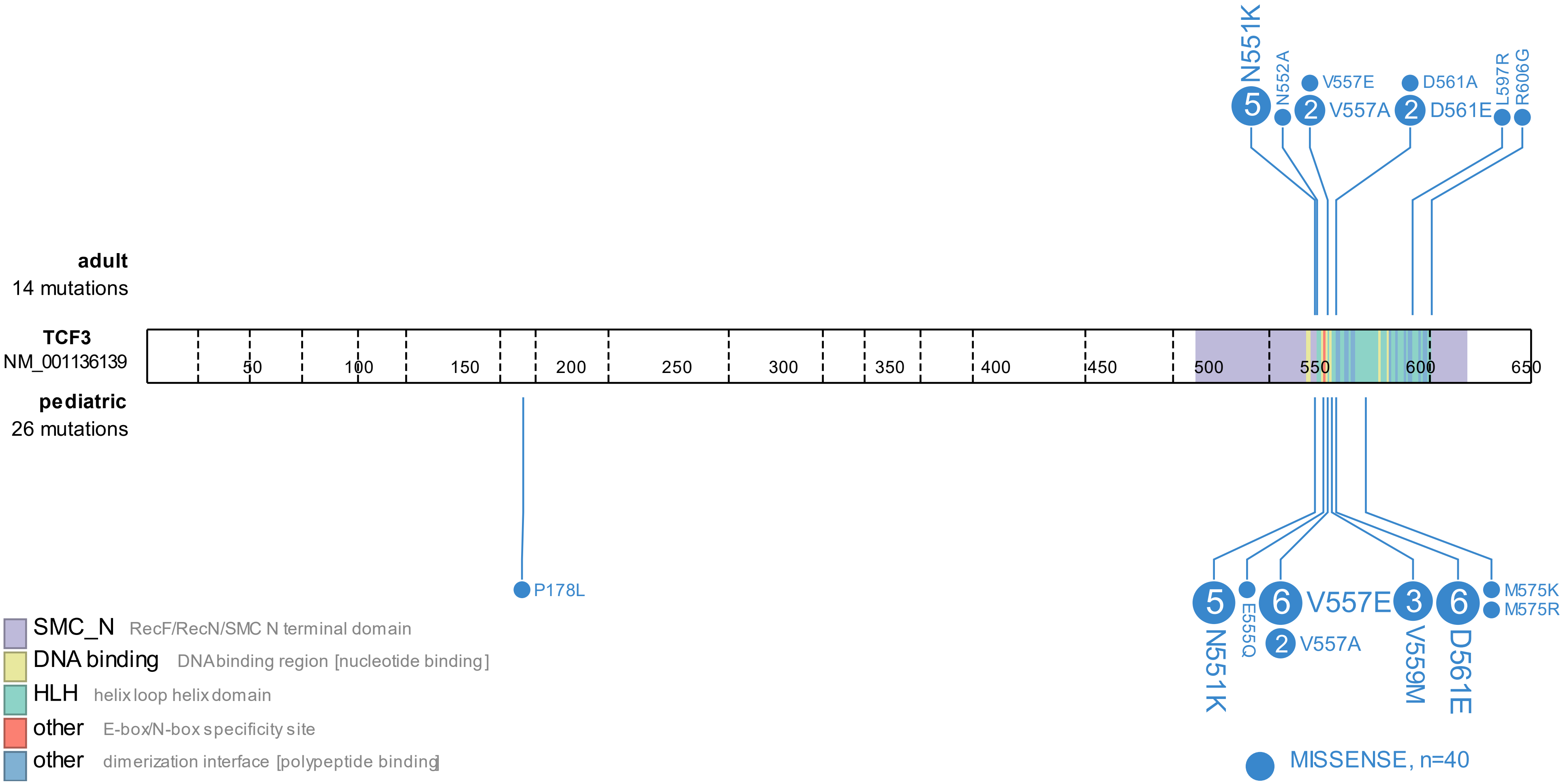

Mutations of TCF3 in adult patients are shown above the protein axis, pediatric cases below.

Supplementary Fig. 5a,b: Overlap of recurrent mutations of the SWI/SNF complex members ARID1A and SMARCA4

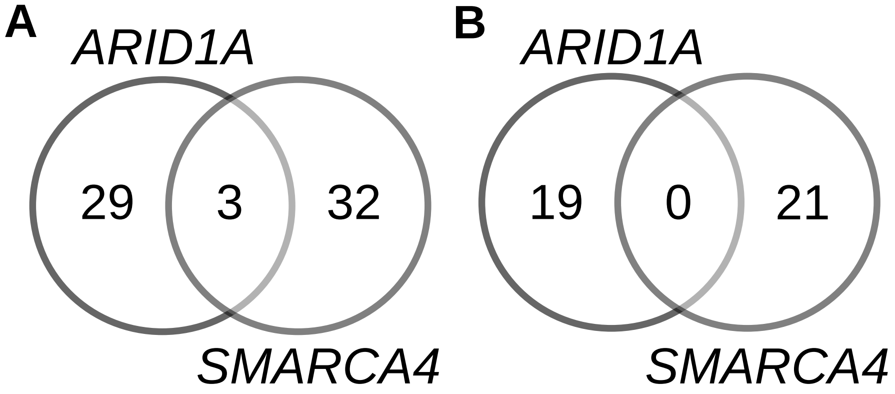

The overlap of recurrent mutations in *ARID1A* and *SMARCA4* is shown for the pediatric (A) and adult subcohort (B) in percent. Taken together, 64% of pediatric cases versus only 40% of adult cases are mutated in any of these genes (p=6e-5, one-tailed Fisher exact test).

Supplementary Fig. 5c: Mutational spectrum of ARID1A

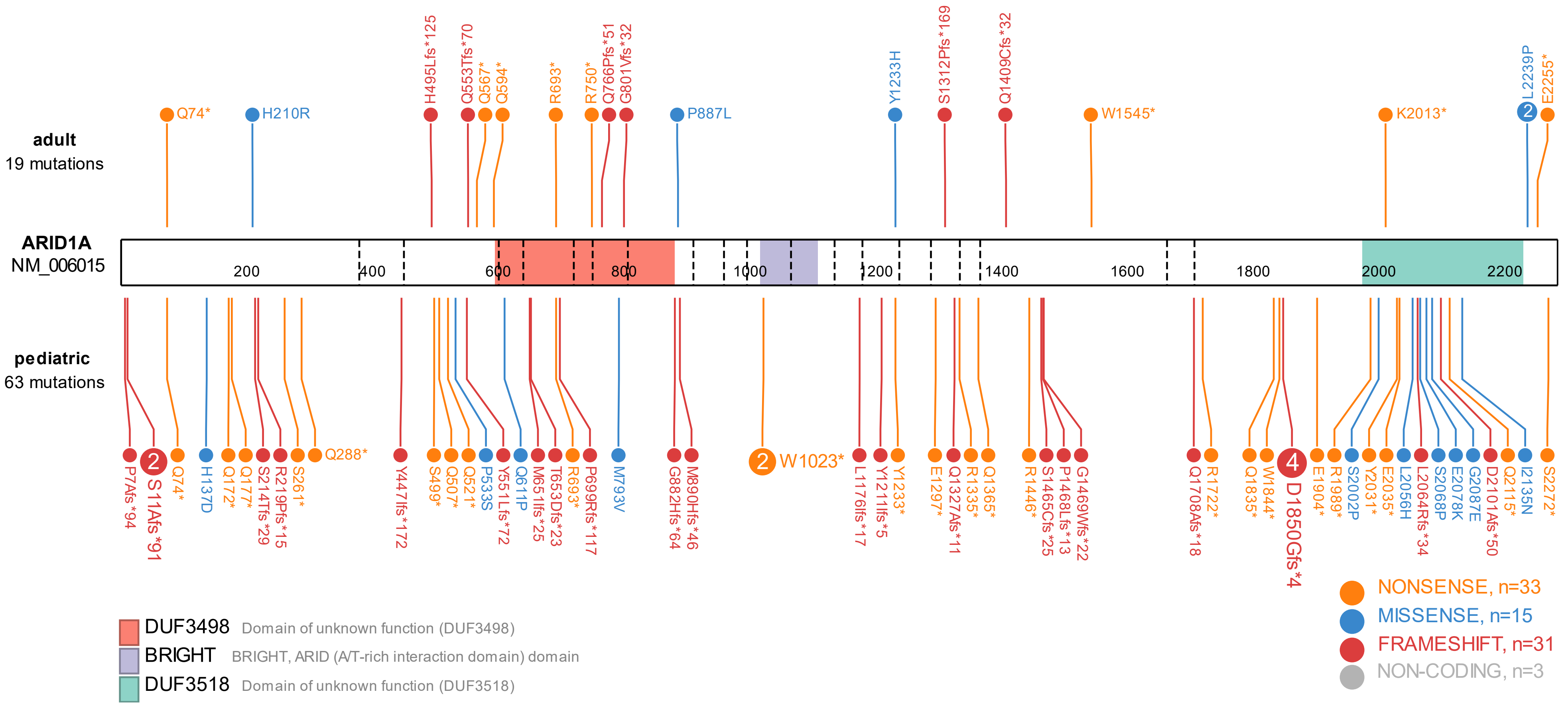

Mutations of ARID1A in adult patients are shown above the protein axis, pediatric cases below.

Supplementary Fig. 5d: Mutational spectrum of SMARCA4

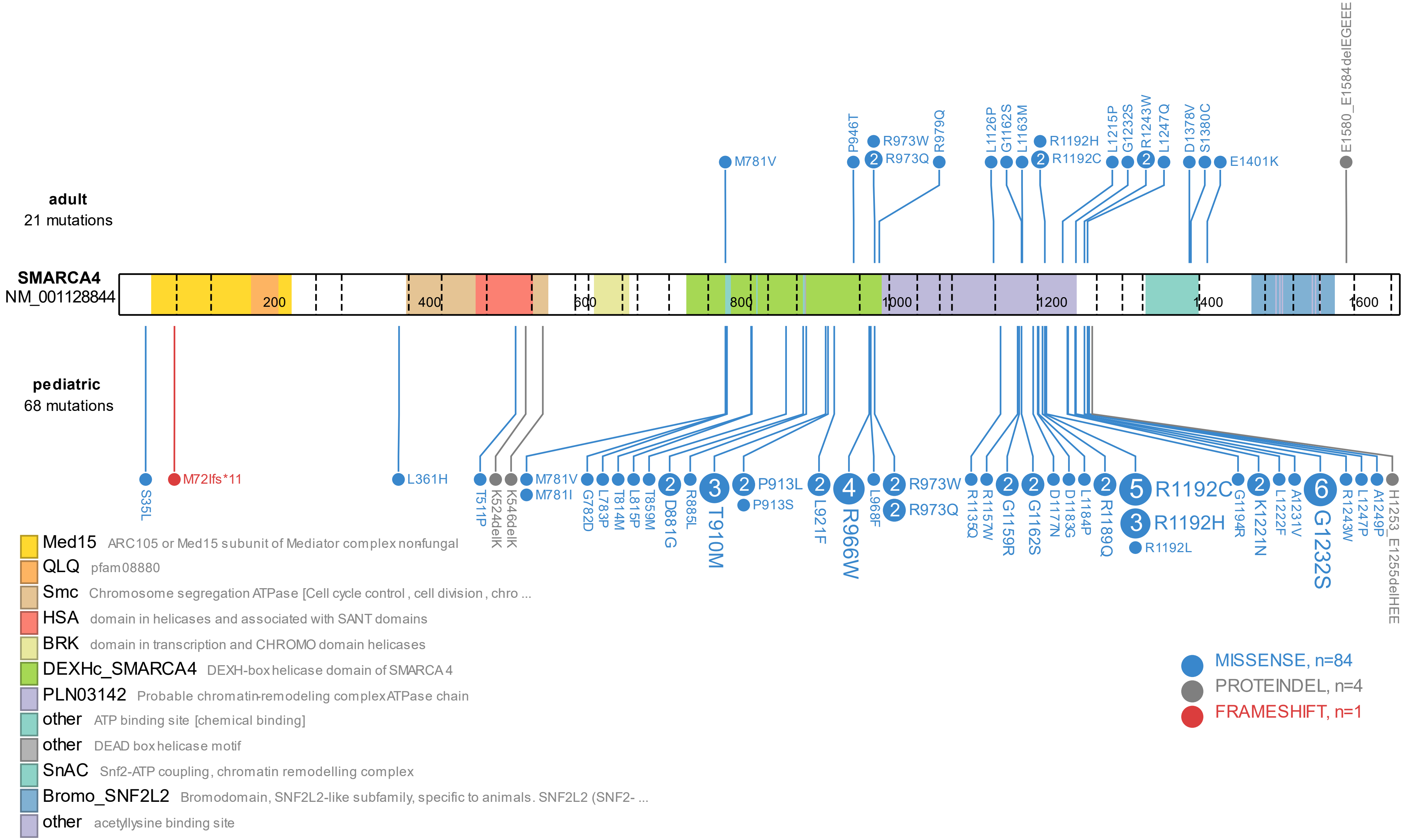

Mutations of SMARCA4 in adult patients are shown above the protein axis, pediatric cases below.

Supplementary Fig. 6a: Mutational spectrum of DDX3X

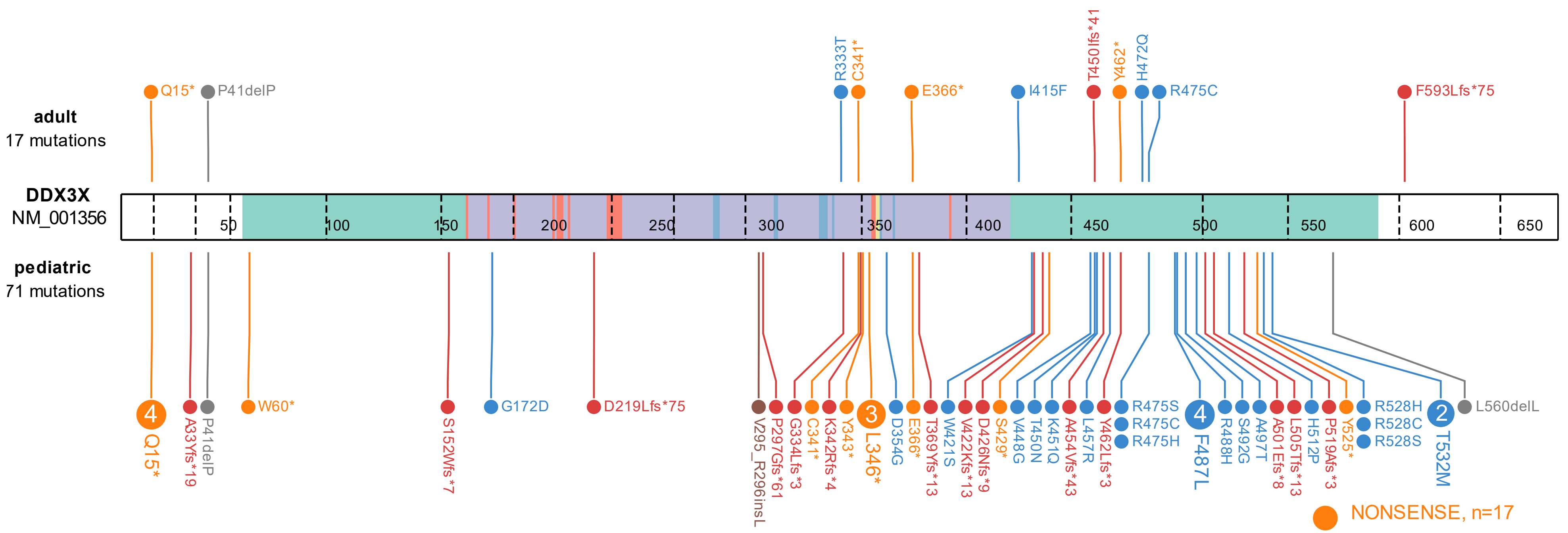

Mutations of DDX3X in adult patients are shown above the protein axis, pediatric cases below.

Supplementary Fig. 6b: Mutational spectrum of GNA13

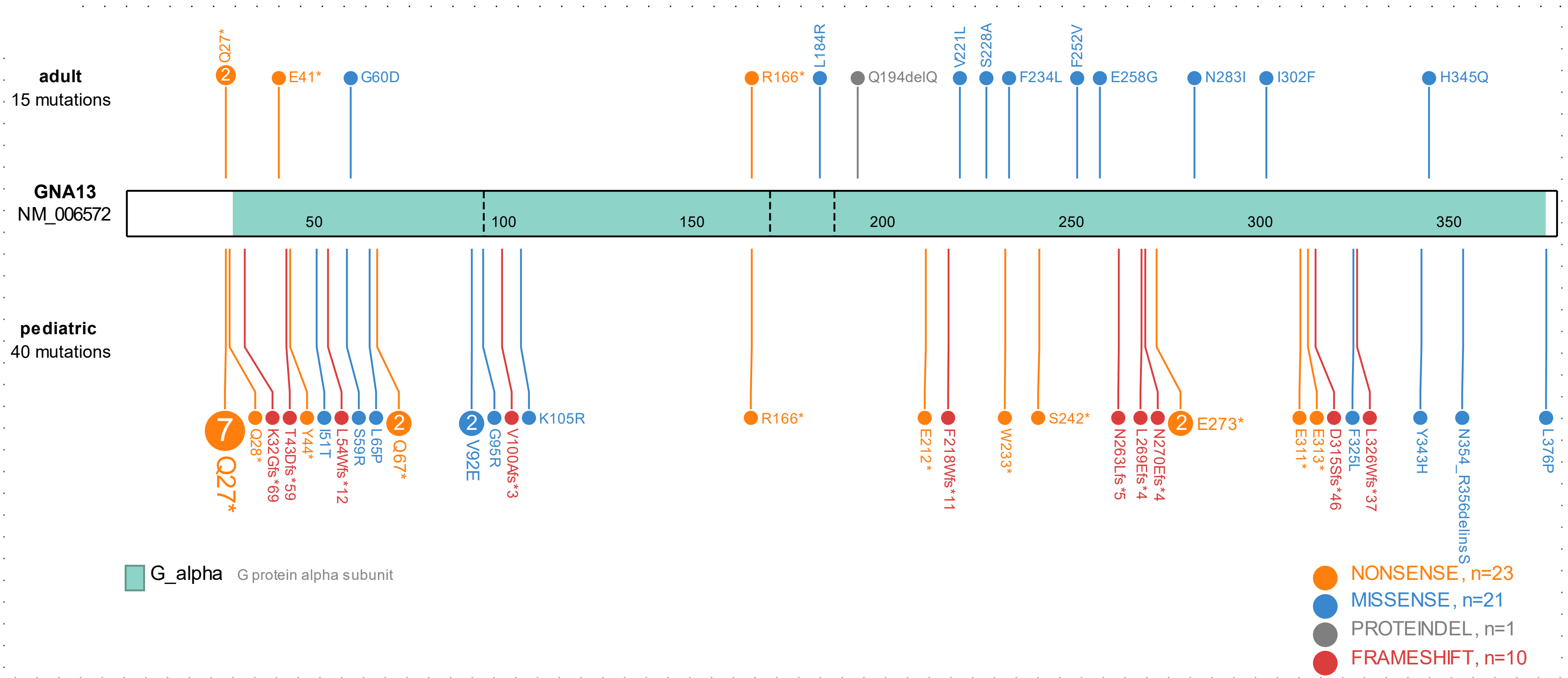

Mutations of GNA13 in adult patients are shown above the protein axis, pediatric cases below.

Supplementary Fig. 6c,d: Overlap of recurrent mutations of GNA13 and P2RY8

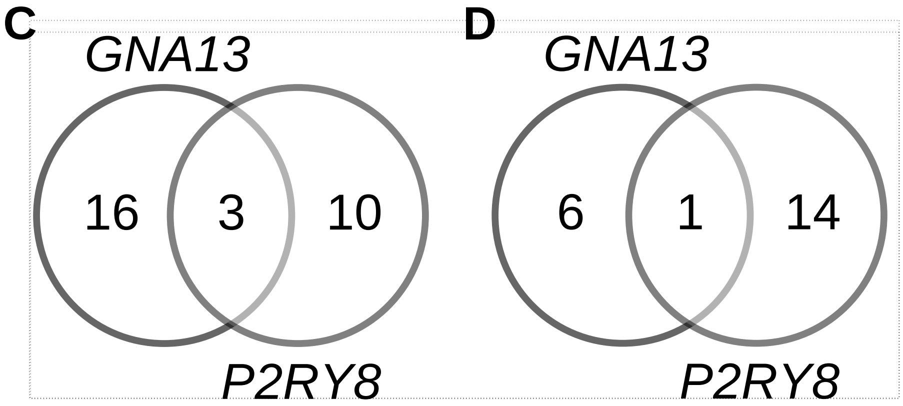

The overlap of recurrent mutations in *GNA13* and *P2RY8* is shown for the pediatric (C) and adult subcohort (D) in percent. Taken together, 29% of pediatric cases versus only 21% of adult cases are mutated in any of these genes (p=0.11, one-tailed Fisher exact test).

Supplementary Fig. 6e: Mutational spectrum of P2RY8

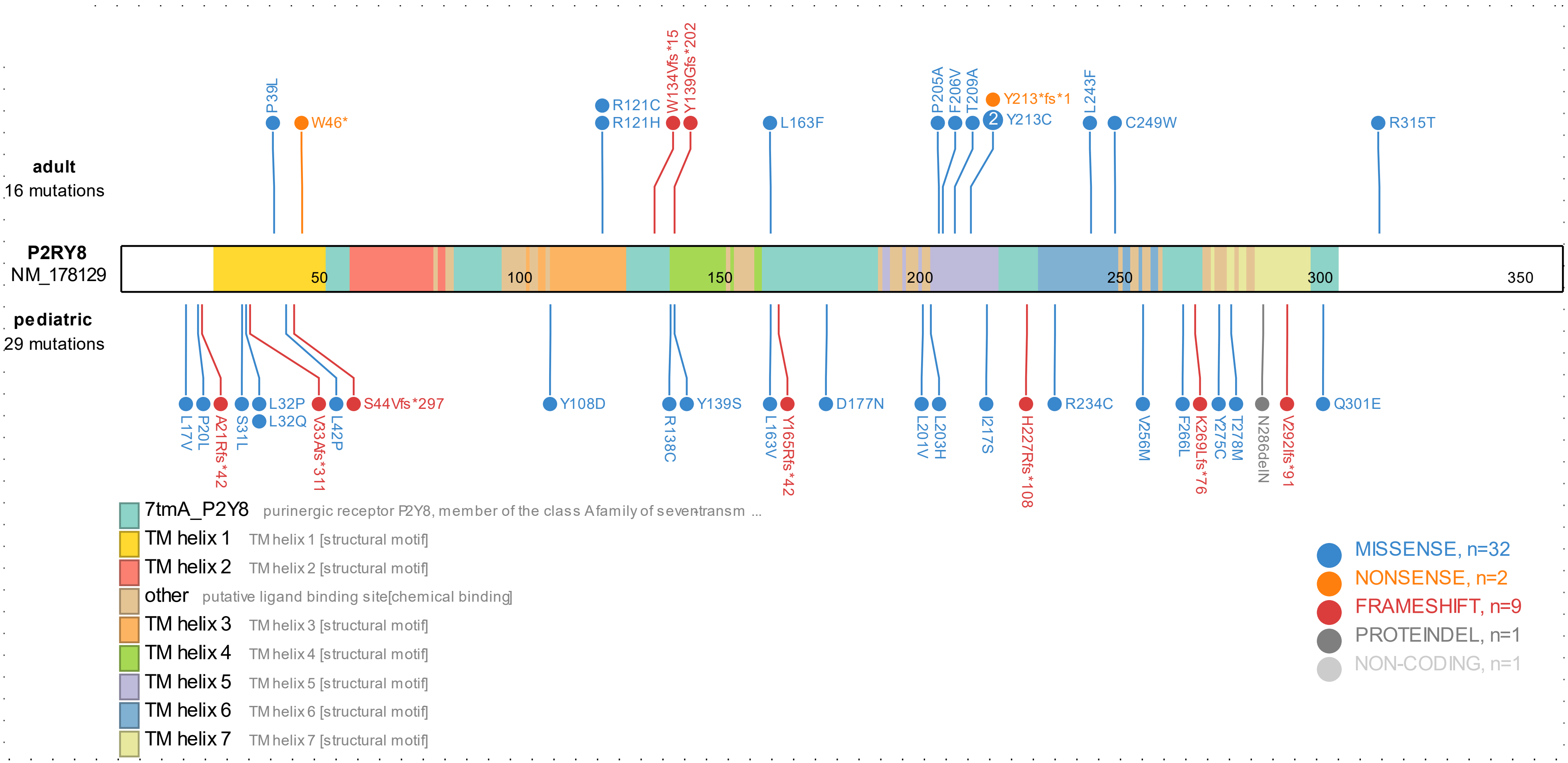

Mutations of P2RY8 in adult patients are shown above the protein axis, pediatric cases below.

Supplementary Fig. 7: Additional associations with outcome in pediatric BL

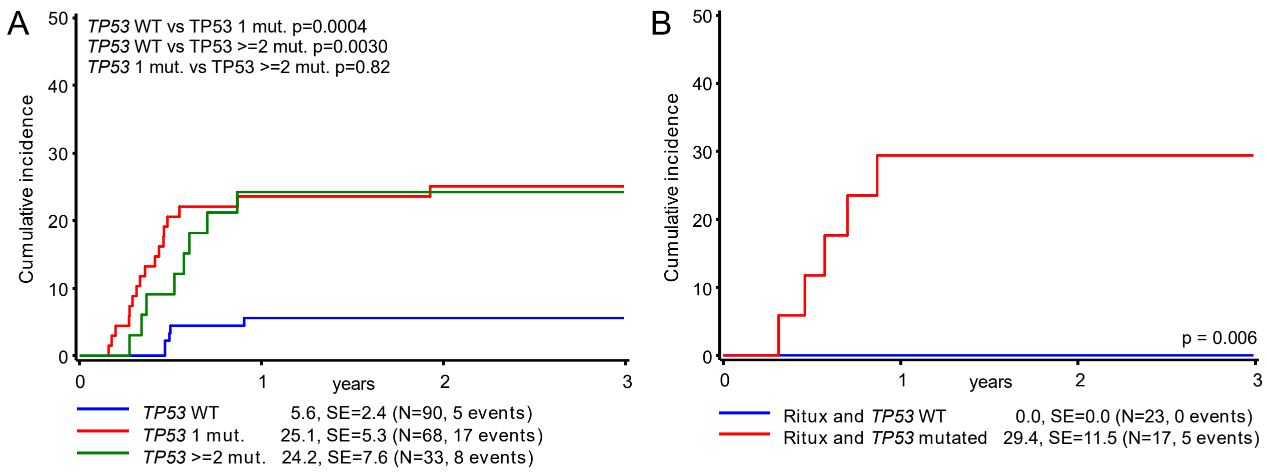

An increased number of *TP53* mutations is not significantly associated with a higher risk of relapse compared to only one *TP53* mutation (A). The significant difference in relapse incidence between wildtype *TP53* and mutated cases is shown for patients treated with rituximab (B).

Supplementary Fig. 8: Overview of available primary BL samples and measurements

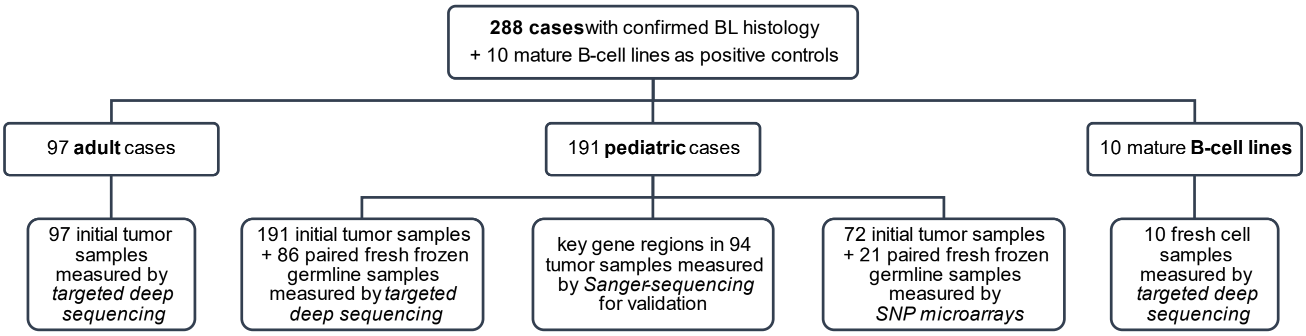

Supplementary Tables

Supplementary Table 1: Overview and outline of clinical characteristics of the pediatric cohort.

Clinical characteristics of pediatric patients diagnosed Burkitt or B-AL (191)		
	**N**	**%**
All	191	100.0%
Male	134	70.2%
Female	57	29.8%
Age <10 years	123	64.4%
Age 10-14 years	50	26.2%
Age >=15 years	18	9.4%
**Stage accoring to St. Jude**		
n.a.	4	2.1%
Stage I + II	18	9.4%
Stage III	74	38.7%
Stage IV/B-AL	95	49.7%
**CNS involvement**		
No	151	79.1%
Yes	40	20.9%
**Bone marrow involvement**		
No	101	52.9%
Yes	90	47.1%
**LDH (U/L) prior start of treatment**		
LDH <500 U/L	40	20.9%
LDH 500-<1000 U/L	40	20.9%
LDH>=1000 U/L	111	58.1%

Data refer to all cases for which the respective information is available.

Supplementary Table 2: Source centers of analyzed samples.

Sources of samples of the pediatric cohort (191 cases)
Department of Pediatric Hematology and Oncology, University Hospital Aachen, Aachen, Germany
Department of Pediatrics, Schwaebisches Kinderkrebszentrum, University Hospital Augsburg, Augsburg, Germany
Department of Pediatric Hematology and Oncology, Charité Campus Virchow-Klinikum, University Hospital Berlin, Berlin, Germany
Department of Pediatric Hematology and Oncology, Evangelisches Klinkum Bielefeld, Bielefeld, Germany
Department of Pediatric Hematology and Oncology, University Hospital Bonn, Bonn, Germany
Department of Pediatric Hematology and Oncology, Staedtisches Klinikum Braunschweig, Braunschweig, Germany
Department of Pediatric Hematology and Oncology, Gesundheit Nord Klinikverbund Bremen, Bremen, Germany
Department of Pediatric Hematology and Oncology, Klinikum Chemnitz, Chemnitz, Germany
Vestische Kinderklink, University Witten/Herdecke, Datteln, Germany
Department of Pediatric Hematology and Oncology, Klinikum Dortmund, Dortmund, Germany
Department of Pediatrics, Carl Gustav Carus TU Dresden, University Hospital Dresden, Germany
Department of Pediatric Hematology and Oncology, University Hospital Duesseldorf, Duesseldorf, Germany
Department of Pediatric Hematology and Oncology, University Hospital Erlangen, Erlangen, Germany
Department of Pediatric Hematology and Oncology, HELIOS Klinikum Erfurt, Erfurt, Germany
Department of Pediatric Hematology and Oncology, University Hospital Essen, Essen, Germany
Department of Pediatric Hematology and Oncology, University Hospital Frankfurt, Frankfurt a.M., Germany
Department of Pediatric Hematology and Oncology, University Hospital Freiburg, Freiburg, Germany
Department of Pediatric Hematology and Oncology, University Hospital Giessen and Marburg, Giessen, Germany
Department of Pediatrics, Georg-August-Universitaet, University Hospital Goettingen, Goettingen, Germany
Department of Pediatric Hematology and Oncology, University Hospital Halle (Saale), Halle-Wittenberg, Germany
Department of Pediatric Hematology and Oncology, University Hospital Hamburg Eppendorf, Hamburg, Germany
Department of Pediatric Hematology and Oncology, Medizinische Hochschule Hannover, Hannover, Germany
Department of Pediatric Hematology and Oncology, University Hospital Heidelberg, Heidelberg, Germany
Department of Pediatric Hematology and Oncology, Gemeinschaftskrankenhaus Herdecke, Herdecke, Germany
Department of Pediatric Hematology and Oncology, University Hospital Homburg Saar, Homburg, Germany
Department of Pediatrics, University Hospital Jena, Jena, Germany
Department of Pediatrics, Staedtisches Klinikum Karlsruhe, Karlsruhe, Germany
Department of Pediatric Hematology and Oncology, Klinikum Kassel Gesundheit Nordhessen, Kassel, Germany
Department of Pediatric Hematology and Oncology, University Hospital Cologne, Cologne, Germany
Department of Pediatric Hematology and Oncology, Kinderkrankenhaus Amsterdamerstrasse, Cologne, Germany
Department of Pediatric Hematology and Oncology, University Hospital Schleswig Holstein, Kiel, Germany
Department of Pediatric Hematology and Oncology, HELIOS Klinikum Krefeld, Krefeld, Germany
Department of Pediatric Hematology and Oncology, University Hospital Leipzig, Leipzig, Germany
Department of Pediatric Hematology and Oncology, University Hospital Schleswig Holstein, Luebeck, Germany
Department of Pediatric Hematology and Oncology, University Hospital Magdeburg, Magdeburg, Germany
Department of Pediatric Hematology and Oncology, Universitätsmedizin der Johannes Gutenberg-Universität Mainz, University Hospital Mainz, Mainz Germany
Department of Pediatric Hematology and Oncology, University Hospital Mannheim, Mannheim, Germany
Department of Pediatric Hematology and Oncology, Dr. von Haunersches-Kinderspital, Klinikum der LMU, Muenchen, Germany
Department of Pediatric Hematology and Oncology, Kinderklinik der TU, Muenchen/Schwabing, Germany
Department of Pediatric Hematology and Oncology, University Hospital Muenster, Muenster, Germany
Department of Pediatric Hematology and Oncology, Cnopf´sche Kinderklinik, Nuernberg, Germany
Department of Pediatric Hematology and Oncology, Klinkum Oldenburg, Oldenburg, Germany
Department of Pediatric Hematology and Oncology, University Hospital Regensburg, Regensburg, Germany

Department of Pediatric Hematology and Oncology, University Hospital Rostock, Rostock, Germany
Department of Pediatric Hematology and Oncology, Asklepios Klinik St. Augustin, St. Augustin, Germany
Department of Pediatric Hematology and Oncology, HELIOS Kliniken Schwerin, Schwerin, Germany
Department of Pediatric Hematology and Oncology, Olgahospital Stuttgart, Stuttgart, Germany
Department of Pediatrics, Klinikum Mutterhaus der Borromaeerinnen, Trier, Germany
Department of Pediatric Hematology and Oncology, University Hospital Tuebingen, Tuebingen, Germany
Department of Pediatric Hematology and Oncology, University Hospital Ulm, Ulm, Germany
Department of Pediatric Hematology and Oncology, University Hospital Wuerzburg, Wuerzburg, Germany
**Sources of samples of the adult cohort (97 cases)**
Center for Genomic and Computational Biology and Department of Medicine, Duke University, Durham, NC, USA
Department for Hematology, Oncology and Tumor Immunology, Corporate Member of Freie Universität Berlin and Humboldt-Universität zu Berlin, Charité - Universitätsmedizin Berlin, Berlin, Germany
Department of Clinical Pathology, Robert-Bosch-Krankenhaus, and Dr. Margarete Fischer-Bosch Institute of Clinical Pharmacology, Stuttgart, Germany
Department of Pathology, University Hospital Münster, Münster, Germany
Institute of Medical Genetics and Pathology, University Hospital Basel, University of Basel, Basel, Switzerland
Institute of Pathology and Neuropathology and Comprehensive Cancer Centre Tübingen, University Hospital Tübingen, Eberhard-Karls-University, Tübingen, Germany
Institute of Pathology, Universität Würzburg and Comprehensive Cancer Centre Mainfranken (CCCMF), Würzburg, Germany
MLL Munich Leukemia Laboratory, Munich, Germany

Supplementary Table 3: Overview of regulated processes and molecular functions from gene ontology that are significantly overrepresented with genes mutated ≥5% (gene set enrichment, one-sided permutation tests)

Go-term	Process	Genes	number	*p*
**43065**	Regulation of apoptotic processes	*DDX3X, FOXO1, ID3, TFAP4, TP53*	5/18 of 8/130	0.0013
**45893**	Positive regulation of transcription, DNA-templated genes	*ARID1A, FOXO1, SMARCA4, TCF3, TFAP4, TP53*	6/18 of 9/130	0.0002
**45892**	Negative regulation of transcription, DNA-templated genes	*CDKN2A, FOXO1, SMARCA4, ID3, TFAP4, TP53*	6/18 of 11/130	0.0009
**Go-term**	**Molecular function**	**Genes**	**number**	***p***
**3713**	Transcription coactivator activity	*ARID1A, SMARCA4, TCF3, TFAP4*	4/18 of 5/130	0.0012
**961**	RNA polymerase II transcription factor activity, sequence-specific DNA binding genes	*FOXO1, TP53*	2/18 of 2/130	0.0182
**8134**	Transcription factor binding genes	*DDX3X, ID3, SMARCA4, TCF3, TP53*	5/18 of 12/130	0.0125

Supplementary Table 4: Selected 134 genes for targeted sequencing

**Panel for targeted sequencing**			
**Gene symbol**			
ABCC5	*DLGAP1*	*LCN15*	*RPL10*
ACAD9	*DOCK4*	*MAP3K6*	*RYR2*
ACE	*DTX1*	*MEF2B*	*SALL3*
ADAMTS5	*E2F2*	*MKI67*	*SBF1*
ADNP	*EDNRB*	*MYC*	*SF3B1*
AGO4	*EHD1*	*MYD88*	*SGK1*
ARHGEF1	*ELP2*	*MYH10*	*SHANK1*
ARID1A	*EML2*	*MYO18A*	*SI*
ATP2C2	*ENKD1*	*NBEAL1*	*SIN3A*
B2M	*ENTPD3*	*NCOR2*	*SLC29A2*
BCL2	*EPHB2*	*NOA1*	*SMARCA4*
BCL6	*ERAP1*	*NOD1*	*SOCS1*
BRAF	*EXOSC6*	*NOTCH1*	*SYNGAP1*
BRD4	*EZH2*	*NRXN2*	*TBC1D9B*
BTG1	*FAM129B*	*P2RY2*	*TCF3*
BTG2	*FBXO11*	*P2RY8*	*TFAP4*
CAD	*FGFR3*	*PC*	*TIGD6*
CARD11	*FLYWCH1*	*PCBP1*	*TLN2*
CCND3	*FOXO1*	*PDCD11*	*TNFAIP3*
CCT6B	*FTCD*	*PIK3C2A*	*TOP2A*
CD79A	*GGTLC1*	*PIK3R1*	*TP53*
CD79B	*GNA13*	*PIM1*	*TPST2*
CDC73	*GNAI2*	*POLRMT*	*TTN*
CDH17	*GRIK5*	*POR*	*UBR4*
CDKN2A	*GTSE1*	*PPP6R2*	*VWA7*
CHD4	*HERC1*	*PREX1*	*WDR90*
COL4A2	*HIST1H1C*	*PRSS22*	*WHAMM*
CPXM2	*HLA-DQB1*	*PTEN*	*YY1AP1*
CREBBP	*ID3*	*PTPRN*	*ZAN*
CXCR4	*ITPR3*	*RANBP6*	*ZBTB7A*
CYB5D1	*KANK2*	*RET*	*ZNF229*
CYP4F22	*KIFC3*	*REV3L*	*ZNF85*
DDX3X	*KLHL26*	*RFX7*	
DHCR7	*KLHL6*	*RHOA*	

Supplementary Table 5: Methods, tools, resources and software used in this study

Method/Tool/Software	Version	Available at	Notes
HISAT2	2.0.4	http://daehwankimlab.github.io/hisat2/download	alignment method
Genome Analysis Toolkit (GATK) / Mutect	4.0.6.0	https://github.com/broadinstitute/gatk/releases	GATK contains Mutect 2 for variant discovery
TransVar	2.4.0 .20180701	https://github.com/zwdzwd/transvar	variant annotator (installed via pip)
dNdScv	0.1.0 20211202	https://github.com/im3sanger/dndscv	cancer driver genes detection
Illumina Genome Studio 2.0	2.0.3	https://sapac.illumina.com/techniques/microarrays/array-data-analysis-experimental-design/genomestudio.html	software suite from SNP array manufracturer
ASCAT	2.4.3	https://github.com/Crick-CancerGenomics/ascat	copy number segmentation and sample purity
GISTIC	2.0	http://portals.broadinstitute.org/cgi-bin/cancer/publications/pub_paper.cgi?mode=view&paper_id=216&p=t	cohort level SNCA analysis (Matlab runtime required)
Integrated Genomics Viewer	2.5.0	http://software.broadinstitute.org/software/igv/download	variant plots in context of their measured reads
Protein Paint	n.a. (web app)	https://pecan.stjude.cloud/proteinpaint	mutation overview plots over protein sequences
GNU parallel	20161222	https://www.gnu.org/software/parallel/	local parallelization of jobs capsuled as bash scripts
samtools	1.10	http://www.htslib.org	sequence file related tasks (manual QC, resorting, indexing)
bedtools	2.27.1	https://bedtools.readthedocs.io	QCs
picard	20180706	https://broadinstitute.github.io/picard/	sequence file related tasks (manual QC, resorting, indexing)
vcfanno	0.2.9	https://github.com/brentp/vcfanno/releases	quickly combine variant annotation sources
Trim Galore!	0.5.0	https://github.com/FelixKrueger/TrimGalore	automation of cutadapt and FastQC iterations
cutadapt	1.16	https://cutadapt.readthedocs.io/en/stable/	preprocessing of sequencing data: cut adapter sequences
FastQC	0.11.5	http://www.bioinformatics.babraham.ac.uk/projects/fastqc	QC of sequencing measurements

Resources/Databases	Version	Available at	Notes
COSMIC	85	https://cancer.sanger.ac.uk/cosmic	resource database of known somatic mutations
NCBI ClinVar	20180429	https://www.ncbi.nlm.nih.gov/clinvar/	resource database of variants with known clinical significance
gnomAD/ExAC	based on v2	provided via GATK resource pack (af-only-gnomad.hg38.ensemble.vcf.gz); created from gnomAD by https://github.com/broadinstitute/gatk/blob/master/scripts/mutect2_wdl/mutect_resources.wdl	resource database of known population germline variants, originally based on ExAC
NCBI Common Human Variants	“common_all.vcf.gz” from 20180418	https://www.ncbi.nlm.nih.gov/variation/docs/human_variation_vcf	resource database of common human variants, part of dbSNP build 151
NCBI RefSeq gene models	20190227	provided via TransVar download; file name hg38.refseq.gff.gz.transvardb; downloaded 20190227	gene models used for advanced variant annotation, e.g. in the codon frame context
APPRIS	20200122	https://appris.bioinfo.cnio.es/#/downloads	principal isoforms/transcripts of genes

Human Reference Genome	GRCh38	http://daehwankimlab.github.io/hisat2/download/#h-sapiens	besides the reference genome, this resource contains indexes for sequence alignment via HISAT2; the identical reference genome was utilized for all downstream analyses

**IDEs and runtimes**	**Version**	**Available at**	**Notes**
MATLAB	R2018a-R2020a	https://www.mathworks.com/pricing-licensing.html?prodcode=ML&intendeduse=edu	general purpose analysis software suite (license required)
Python	2.7 and 3.6	https://www.python.org/	general purpose language and runtime (needed by TransVar)
R	3.6.3	https://www.r-project.org/	Statistics analysis language and runtime (needed by ASCAT)

Supplementary Table 6: Selected primers used for Sanger sequencing

Target	Primer for Sanger sequencing
CCND3 Exon5 F1	CCATGTGTTGGGAGCTGTC
CCND3 Exon5 R1	CTGGAGGCAGGGAGGTG
FBXO11 Exon 15/16 F2	GCCATTACCTCCTTACTCGG
FBXO11 Exon 15/16 R2	CAGTGGCTTCTGTCCTCACC
FBXO11 Exon 17/18 F1	ATGGTGGTCTGGAACGGAAC
FBXO11 Exon 17/18 R1	GGGCAGGCACTATGGAACTT
FBXO11 Exon 22/23 F2	TCCCTCCGAAGACACAGATG
FBXO11 Exon 22/23 R2	CCCAGCTTTGAGATCCTGAGT
FBXO11 Exon 6/7 F1	TGTTTCTGGATATTACCGGATCA
FBXO11 Exon 6/7 R1	CGGGGAGAAAGAATTTATATACAGC
FOXO1 Exon1 F1	GTCCGTCCTTCCGTCCG
FOXO1 Exon1 R1	CACGCTCTTGACCATCCACT
ID3 Exon1-2 F1	ATTTAAGCGGGCTGTGAACG
ID3 Exon1-2 R1	TTTGATGCAACCATGGGCAA
P2RY8 Exon 1 F1	CCTGCTAAAGCATCTCCCCC
P2RY8 Exon 1 R1	TGCCTGGGAGGAATAAAGCC
PCBP1 F1	TCCCTGATTGGGAAAGGCGG
PCBP1 R1	CGCGCAGATGACTGGGGAGC
TCF3 Exon18 F2	TTTACAAGCCTCCACCCCAT
TCF3 Exon18 R2	CCCATCACTCCGAACCTTGT
TP53 Exon 1/2 F3	CTGAGGTGTAGACGCCAACT
TP53 Exon 1/2 R3	ACTTTGCACATCTCATGGGG

List of Supplementary Data

Supplementary Data 1: Cohort overview

Supplementary Data 2: Called somatic mutations (summarized by Fig.1).

Supplementary Data 3: Variant filtering overview and statistics of the multi-stage filter hierarchy

Supplementary Data 4: Validation of discovered somatic mutations by Sanger sequencing

Supplementary Data 5: Identification of cancer genes by mutation abundance using dN/dS

Supplementary Data 6: Discovered recurrent somatic copy number aberrations (GISTIC analysis)

Supplementary Data 7: Discovered copy number aberrations on gene level (ASCAT analysis)

Supplementary Data 8: Subcohort comparisons (summarized by Figs. 3 and 4).

Supplementary Data 9: Hotspot analysis summary for pediatric and adult samples
