# Supplementary material for: Clinical relevance of molecular characteristics in Burkitt lymphoma differs according to age
Source: Nat Commun. 2022 Jul 6;13:3881. doi: 10.1038/s41467-022-31355-8 (PMC9259584; doi:10.1038/s41467-022-31355-8)
Supplement: Supplementary file 3 — Description of Additional Supplementary Files [file 41467_2022_31355_MOESM3_ESM.pdf]

Description of Additional Supplementary Files

File Name: Supplementary Data 1
Description: Cohort overview

File Name: Supplementary Data 2
Description: Called somatic mutations (summarized by Fig.1).

File Name: Supplementary Data 3
Description: Variant filtering overview and statistics of the multi-stage filter hierarchy.

File Name: Supplementary Data 4
Description: Validation of discovered somatic mutations by Sanger sequencing.

File Name: Supplementary Data 5
Description: Identification of cancer genes by mutation abundance using dN/dS.

File Name: Supplementary Data 6
Description: Discovered recurrent somatic copy number aberrations (GISTIC analysis).

File Name: Supplementary Data 7
Description: Discovered copy number aberrations on gene level (ASCAT analysis).

File Name: Supplementary Data 8
Description: Subcohort comparisons (summarized by Figs. 3 and 4).

File Name: Supplementary Data 9
Description: Hotspot analysis summary for pediatric and adult samples.
