# Supplementary material for: Clinical relevance of molecular characteristics in Burkitt lymphoma differs according to age
Source: Nat Commun. 2022 Jul 6;13:3881. doi: 10.1038/s41467-022-31355-8 (PMC9259584; doi:10.1038/s41467-022-31355-8)
Supplement: Supplementary file 13 — Reporting Summary [file 41467_2022_31355_MOESM13_ESM.pdf]

nature portfolio

Corresponding author(s): Birgit Burkhardt

Last updated by author(s): Jun 9, 2022

Reporting Summary

Nature Portfolio wishes to improve the reproducibility of the work that we publish. This form provides structure for consistency and transparency in reporting. For further information on Nature Portfolio policies, see our Editorial Policies and the Editorial Policy Checklist.

Statistics

For all statistical analyses, confirm that the following items are present in the figure legend, table legend, main text, or Methods section.

n/a	Confirmed	
☐	☒	The exact sample size (*n*) for each experimental group/condition, given as a discrete number and unit of measurement
☐	☒	A statement on whether measurements were taken from distinct samples or whether the same sample was measured repeatedly
☐	☒	The statistical test(s) used AND whether they are one- or two-sided<br>*Only common tests should be described solely by name; describe more complex techniques in the Methods section.*
☐	☒	A description of all covariates tested
☐	☒	A description of any assumptions or corrections, such as tests of normality and adjustment for multiple comparisons
☐	☒	A full description of the statistical parameters including central tendency (e.g. means) or other basic estimates (e.g. regression coefficient) AND variation (e.g. standard deviation) or associated estimates of uncertainty (e.g. confidence intervals)
☐	☒	For null hypothesis testing, the test statistic (e.g. *F*, *t*, *r*) with confidence intervals, effect sizes, degrees of freedom and *P* value noted<br>*Give P values as exact values whenever suitable.*
☒	☐	For Bayesian analysis, information on the choice of priors and Markov chain Monte Carlo settings
☒	☐	For hierarchical and complex designs, identification of the appropriate level for tests and full reporting of outcomes
☒	☐	Estimates of effect sizes (e.g. Cohen's *d*, Pearson's *r*), indicating how they were calculated

Our web collection on statistics for biologists contains articles on many of the points above.

Software and code

Policy information about availability of computer code

Data collection	Metadata were collected and stored as Microsoft Excel (v2016-v365) files and preprocessed with MATLAB R2018a-R2020a.
Data analysis	We used the following software programs and packages (in lexical order): ASCAT 2.4.3 [https://github.com/Crick-CancerGenomics/ascat], bedtools 2.27.1 [https://bedtools.readthedocs.io], cutadapt 1.16 [https://cutadapt.readthedocs.io/en/stable/], dNdScv 0.1.0 (20211202) [https://github.com/im3sanger/dndscv], FastQC 0.11.5 [http://www.bioinformatics.babraham.ac.uk/projects/fastqc], Genome Analysis Toolkit (GATK) / Mutect 4.0.6.0 [https://github.com/broadinstitute/gatk/releases], GISTIC 2.0 [https://github.com/broadinstitute/gistic2], GNU parallel 20161222 [https://www.gnu.org/software/parallel/], HISAT2 2.0.4 [http://daehwankimlab.github.io/hisat2/download], Illumina Genome Studio 2.0.3 [https://sapac.illumina.com/techniques/microarrays/array-data-analysis-experimental-design/genomestudio.html], Integrated Genomics Viewer 2.5.0-2.8.0 [http://software.broadinstitute.org/software/igv/download], MathWorks MATLAB R2018a-R2020a [https://www.mathworks.com], picard 20180706 [https://broadinstitute.github.io/picard/], Protein Paint web app [https://pecan.stjude.cloud/proteinpaint], Python 2.7 and 3.6 [https://www.python.org], R 3.6.3 [https://www.r-project.org], samtools 1.1 [http://www.htslib.org], TransVar 2.4.0 (20180701) [https://github.com/zwdzwd/transvar], Trim Galore! 0.5.0 [https://github.com/FelixKrueger/TrimGalore], and vcfanno 0.2.9 [https://github.com/brentp/vcfanno/releases]. See Supplementary Figure 1 for a schematic overview of main analyses. Detailed descriptions of our analyses are provided in methods and Supplementary Information. Supplementary Table 5 provides additional details on tool availabilities.

For manuscripts utilizing custom algorithms or software that are central to the research but not yet described in published literature, software must be made available to editors and reviewers. We strongly encourage code deposition in a community repository (e.g. GitHub). See the Nature Portfolio guidelines for submitting code & software for further information.

Data

Policy information about availability of data

All manuscripts must include a data availability statement. This statement should provide the following information, where applicable:

- Accession codes, unique identifiers, or web links for publicly available datasets
- A description of any restrictions on data availability
- For clinical datasets or third party data, please ensure that the statement adheres to our policy

The targeted sequencing data EGAD00001007708 in FASTQ.gz format [https://ega-archive.org/datasets/EGAD00001007708] and SNP data EGAD00010002137 in IDAT format [https://ega-archive.org/datasets/EGAD00010002137] generated in this study have been deposited in the European Genome-phenome Archive (EGA) under study accession EGAS00001005270 [https://ega-archive.org/studies/EGAS00001005270]. These data are available under restricted access for German data privacy laws; access can be obtained via the associated data access committee EGAC00001002105 [https://ega-archive.org/dacs/EGAC00001002105]. EGA access will be granted after a data access treaty has been agreed upon with the law department of the University Hospital Muenster. Restrictions include limiting access to those people named in the agreement for the duration of the named project. Typically, access for universities or public research institutions is granted within one month if there are no amendments from your side. The processed somatic mutations and copy number aberrations as well as clinical metadata are provided in respective Supplementary Data items. The following public data sources were used in this study: The human reference genome from the Genome Reference Consortium (GRCh38) in its pre-indexed form for alignment with HISAT2 [http://daehwankimlab.github.io/hisat2/download/#h-sapiens], the Catalogue Of Somatic Mutations In Cancer (COSMIC, v85) [https://cancer.sanger.ac.uk/cosmic], the NCBI database of common human variants (based on dbSNP build 151, version 2018-04) [https://www.ncbi.nlm.nih.gov/variation/docs/human_variation_vcf]), NCBI ClinVar (version 2018-04) [https://www.ncbi.nlm.nih.gov/clinvar/], NCBI RefSeq gene models via TransVar (file name hg38.refseq.gff.gz.transvardb, downloaded 20190227), gnomAD/ExAC germline variants as provided in the file af-only-gnomad.hg38.ensemble.vcf.gz of the GATK resource bundle originally accessed via ftp.broadinstitute.org/bundle, but since moved by the Broad Institute to Google cloud bucket; see [https://gatk.broadinstitute.org/hc/en-us/articles/360035890811-Resource-bundle] for access information, and the principal splice isoforms database (APPRIS, version 2020-01-22) [https://appris.bioinfo.cnio.es/#/downloads]. All remaining data is available within the Article Supplementary Information and Supplementary Datasets.

Field-specific reporting

Please select the one below that is the best fit for your research. If you are not sure, read the appropriate sections before making your selection.

☒ Life sciences ☐ Behavioural & social sciences ☐ Ecological, evolutionary & environmental sciences

For a reference copy of the document with all sections, see nature.com/documents/nr-reporting-summary-flat.pdf

Life sciences study design

All studies must disclose on these points even when the disclosure is negative.

Sample size	For this retrospective study of the molecular characteristics of Burkitt Lymphoma and its clinical relevance in different age groups, no sample size or power calculation was performed. Instead, the maximum number of obtainable samples were collected. For composition of this cohort, see Supplementary Data 1.<br>Additionally, analysis of 10 Burkitt Lymphoma cell lines was performed.
Data exclusions	Pre-established criteria for data exclusion:<br>1. Obtain sufficient DNA to analyze samples.<br>2. Obtain sufficient quality control in these analyses.<br>For 396 targeted samples (patients and cell lines, 298 initial tumors from 288 patients and 10 cell lines, 98 normals) we could extract sufficient DNA to perform targeted resequencing. 9 samples were excluded after alignment, pairing and coverage QC (outliers with too few mapped reads, relative to the distribution of mapped reads in all samples). For 93 BL samples (21 normals, 72 initial tumors) we obtained sufficient DNA to perform SNP arrays. All samples passed ASCAT QC (pre-established binary quality estimate in the ASCAT algorithm).
Replication	Next-generation sequencing was performed for 134 genes in 298 samples. Validation by sanger sequencing was performed in a subset of 191 pediatric patient samples and cell lines. The genes ID3, CCND3 and TCF3 were sequenced in 72 samples. In case of a mutation the result was confirmed in a second experiment. TP53 was sequenced in 57 samples, FOXO1 and FBXO11 in 25, PCBP1 in 13 and P2RY8 in 12 samples. In case of inconsistence with data from targeted DNA-sequencing the result was confirmed in a second experiment. Two variants discovered by Sanger-sequencing were not rediscovered by targeted DNA-sequencing. Three of the somatic SNV/indels called by targeted DNA-sequencing were not part of the Sanger validation set. This results in a sensitivity of 0.9943 and a specificity 0.9925 for targeted DNA-sequencing.<br>SNP array experiments were performed in 72 samples for which sufficient material was available. Due to scarcity of material in the majority of cases, replication was not performed.
Randomization	This is a retrospective study. Therefore randomization was not possible.<br>Regarding the analysis of outcome in pediatric patients: there are known risk factors for survival like LDH or stage of disease which could be used as covariates in a multivariate analysis. Since we wanted to explore the genetic backround of relapse it is not useful to include these factors which are secondary to the underlying genetics.
Blinding	We investigated molecular characteristics of lymphoma samples and correlation to clinical characteristics as retrieved from the study centres databank. As this is a retrospective exploratory study blinding was not applicable.

Reporting for specific materials, systems and methods

We require information from authors about some types of materials, experimental systems and methods used in many studies. Here, indicate whether each material, system or method listed is relevant to your study. If you are not sure if a list item applies to your research, read the appropriate section before selecting a response.

Materials & experimental systems

n/a	Involved in the study
☒	☐ Antibodies
☐	☒ Eukaryotic cell lines
☒	☐ Palaeontology and archaeology
☒	☐ Animals and other organisms
☐	☒ Human research participants
☒	☐ Clinical data
☒	☐ Dual use research of concern

Methods

n/a	Involved in the study
☒	☐ ChIP-seq
☒	☐ Flow cytometry
☒	☐ MRI-based neuroimaging

Eukaryotic cell lines

Policy information about cell lines

Cell line source(s)	Cell lines Blue-1, Daudi, Dogkit, Gumbus, Jijoye, Raji and Ramos were obtained from Louis Staudt (National Cancer Institute, Bethesda) and authenticated by SNP typing. Namalwa and BL-60 were obtained from Stephan Mathas (Max Delbrueck Center, Berlin) and authenticated by SNP typing. BL-70 was obtained from the DSMZ, Braunschweig, Germany (catalog number ACC 233).
Authentication	Cell lines BL-60, Blue-1, Daudi, Dogkit, Gumbus, Jijoye, Namalwa, Raji, Ramos were authenticated by SNP typing (Multiplexion, Heidelberg, Germany). BL-70 was not authenticated since it was obtained directly from the DSMZ.
Mycoplasma contamination	all cell lines were tested negative for mycoplasma contamination
Commonly misidentified lines (See ICLAC register)	no commonly misidentified cell line was used in this study

Human research participants

Policy information about studies involving human research participants

Population characteristics	Tumor material from 191 pediatric and 97 adult primary Burkitt lymphoma patients was analyzed. Cases with 11q aberration have been excluded, as they are considered a different entity. There were no other prerequisites concerning age, gender, stage of disease or health status. For the 191 pediatric cases, the median age at diagnosis was 9 years. 30% of pediatric patients were female. Approximately 10% were diagnosed with stage I/II disease, according to the St. Jude staging system, while about 40% had stage III and 50% stage IV disease. The clinical characteristics of the pediatric cohort are summarized in Supplementary Table 1. The adult cohort comprised 97 cases, with a median age of 52 years at diagnosis, and was 40% female (Supplementary Data 1).
Recruitment	All pediatric patients were participants in one of the consecutive uniform treatment protocols NHL-BFM95, B-NHL BFM04 or NHL-BFM Registry 2012. Patients were initially recruited by the treating pediatric cancer center and included in the protocol after confirmation of the diagnosis by central reference laboratories. Material for the present study was used only, if sufficient excess material was available in the study centres biobank after completion of diagnostics.<br>Material from adult patients was collected from collaborating experienced laboratories if sufficient material was available after completion of diagnostics.<br>Since we collected cases with sufficient obtainable material, there is no self-selection bias.
Ethics oversight	Written informed consent was obtained from all patients and/or their legal guardians. Patients did not receive any financial compensation for participation. This study was approved by the ethics committee of the medical association Westfalen-Lippe, Germany and the University of Muenster, Germany (pediatric cohort: 2015-495-f-S, adult cohort: 2017-534-f-S).

Note that full information on the approval of the study protocol must also be provided in the manuscript.
